# Seeing the Unseen: Illustrated Reflection as a Tool for Risk Awareness in Surgical Training

**DOI:** 10.7759/cureus.104855

**Published:** 2026-03-08

**Authors:** Loc H Tran

**Affiliations:** 1 General Surgery Department, School of Medicine, University of Medicine and Pharmacy at Ho Chi Minh City, Ho Chi Minh City, VNM; 2 Digestive Surgery Department, Nhan Dan Gia Dinh Hospital, Ho Chi Minh City, VNM

**Keywords:** appendectomy, drawing, education, internship and residency, laparoscopy

## Abstract

The transition from theoretical knowledge to operative performance represents a pivotal stage in surgical training. Although trainees may accurately reproduce the visible technical steps demonstrated by supervisors, the underlying operative reasoning and risk anticipation are not always explicitly conveyed. Decisions regarding dissection planes, energy application, and tissue handling often reflect implicit experience-based judgment. This gap between observable action and internal reasoning may limit the development of independent surgical thinking and increase the likelihood of preventable technical errors during early operative training. This technical report proposes drawing as an active reflective tool to enhance risk awareness in surgical education. Using mesoappendix division during laparoscopic appendectomy as an illustrative example, the PRAI (Purpose-Risk-Approach-Illustration) framework is introduced to guide structured stepwise reflection. Through simplified anatomical reconstruction, trainees identify essential structures, delineate risk zones, and clarify the relationship between operative objectives and technical strategy. Drawing in this context functions not merely as a visual aid but as a cognitive process that transforms operative experience into structured spatial understanding. By reconstructing operative steps through illustration, trainees may strengthen spatial orientation, anticipate complications more effectively, and transition from imitative performance toward analytical reasoning. Importantly, illustrated reflection is not intended to replace established reflective practices such as operative debriefing, operative note review, or cognitive task analysis, but rather to serve as a complementary structured reflective tool. Drawing may also create cognitive “memory anchors” that facilitate spatial encoding and retention. Future research may evaluate its educational impact using established assessment tools, including Objective Structured Assessment of Technical Skills (OSATS) domains such as respect for tissue, instrument handling, and procedural flow; structured surgical error taxonomies (e.g., incorrect dissection plane or inappropriate energy use); near-miss event documentation such as bleeding from premature vessel division or potential thermal injury to adjacent bowel; time to identification of critical anatomical structures (e.g., the appendiceal artery); and cognitive workload scales such as the National Aeronautics and Space Administration Task Load Index (NASA-TLX). Such metrics may allow quantitative evaluation of whether illustrated reflection improves operative judgment and technical safety.

## Introduction

The transition from theoretical study of anatomy, operative principles, and preclinical simulation to live surgery marks a critical stage in surgical training. Foundational knowledge provides structure, but operative environments introduce variability, limited visualization, and time-dependent decision-making that cannot be fully replicated outside the operating room [1]. Clinical performance requires the integration of anatomical knowledge with real-time judgment. This shift places new cognitive demands on trainees beyond technical execution alone.

Observation and imitation are common in early training. Trainees often reproduce visible technical steps demonstrated by supervisors. However, operative actions are guided by internal reasoning that is not always verbalized. Decisions regarding dissection planes, energy application, and tissue handling reflect applied anatomical interpretation shaped by experience [2]. As a result, trainees may replicate movements without fully understanding the underlying risk considerations. This discrepancy between visible action and implicit reasoning may limit the development of independent judgment.

Drawing provides visual memory anchors that may be more durable than written notes alone [3]. When trainees reconstruct an operative step through simplified illustration, they create a structured representation of spatial relationships and key anatomical landmarks. The process of selecting essential structures and identifying areas at risk requires active cognitive processing. This form of visual encoding has been shown to enhance recall in experimental settings [3] and may therefore support situational awareness during operative procedures.

Structured reflection extends beyond memory reinforcement. By revisiting moments where risk was insufficiently anticipated or judgment was incomplete, trainees may reduce the likelihood of repeating similar technical oversights. Visual reconstruction makes potential hazards explicit and promotes anticipatory rather than reactive thinking [4]. In this way, illustrated reflection may contribute to safer operative behavior by fostering greater risk awareness and reducing the recurrence of preventable technical errors.

This technical report describes a structured and illustrated reflection method for surgical training, illustrated through an example of mesoappendix division during laparoscopic appendectomy.

## Technical report

Structured reflection using a framework

Reflection is useful in surgery when it is directly connected to anatomical relationships, operative steps, and risk control. Rather than describing a procedure in general terms, illustrated reflection in this report is used to examine a specific operative step in detail. The choice of reflective structure may vary according to individual preference and educational context. The PRAI framework was primarily derived from practical observations during surgical training. In particular, it arose from the need to strengthen patient safety awareness and to support trainees in developing independent operative judgment after a period of close supervision. The framework was also motivated by the noticeable variability in learning progression among trainees. These observations encouraged the development of a simple reflective structure to help trainees systematically analyze operative steps, risks, and decision-making. This structure was subsequently termed the PRAI (Purpose-Risk-Approach-Illustration) framework and is applied as a practical stepwise sequence, as summarized in Table 1.

**Table 1:** PRAI framework. PRAI: Purpose-Risk-Approach-Illustration. Source: Developed by the author.

Steps of the PRAI framework	Question
Purpose	What is the objective of this operative step?
Risk	What complications may occur if the step is performed improperly?
Approach	What is the optimal technical strategy to achieve the purpose while minimizing risk?
Illustration	How can the step and its risks be visualized to reinforce understanding?

In practice, trainees participating in surgical procedures are encouraged to select an operative step that they find particularly meaningful or technically challenging. Approximately one reflective PRAI entry is completed every three days using a one-page template, resulting in around 10 reflections per month. Each entry focuses on a single operative step and requires trainees to define the objective, identify potential complications, outline a technical plan, and reconstruct the reasoning visually through a simplified illustration. In this way, the drawing serves a functional rather than an aesthetic purpose; it externalizes operative thinking and allows technical decisions to be reviewed clearly. These reflections are documented using a structured PRAI reflection form, as presented in Table 2, and are discussed with supervising surgeons during monthly review sessions. This simple workflow may facilitate consistent implementation across training programs while remaining feasible in busy clinical environments.

**Table 2:** PRAI reflective template for operative steps. PRAI: Purpose-Risk-Approach-Illustration. Source: Developed by the author.

Reflective content		
Date	e.g., DD/MM/YYYY – DD/MM/YYYY	
Surgical procedure		
Operative step		
Purpose		
Risk		
Approach		
Illustration	(Insert Figure 1) and (Insert Figure 2)	
Supervisor’s feedback		Signature
Revisions or additions by the trainee		

Application of the PRAI framework to a selected operative step

The surgical procedure is first divided into discrete steps. A specific step requiring deeper reflection is selected. In this example, we illustrate mesoappendix division and appendiceal artery control during laparoscopic appendectomy. This step was selected because the mesoappendix division can be technically demanding for trainees. Anatomical variation and careful recognition of potential intraoperative risks further increase its complexity [5].

Within this selected step, the PRAI framework was applied through four integrated components. First, the purpose was defined as complete mesoappendix division with secure appendiceal artery control to prevent bleeding and maintain a stable operative field. Second, the risk was identified as hemorrhage from inadequate vascular sealing, thermal injury to the adjacent small bowel due to imprecise energy application, and incomplete mesoappendix division resulting in residual tissue. Third, the approach emphasized deliberate exposure of the operative field, protection of surrounding bowel loops, identification of an appropriate division line near the appendiceal base, and controlled energy application before transection to ensure reliable vascular control. Finally, the illustration translated these structured considerations into a simplified visual representation, allowing operative reasoning to be examined in a systematic manner.

Stepwise illustrated surgical reflection framework

**Figure 1:**
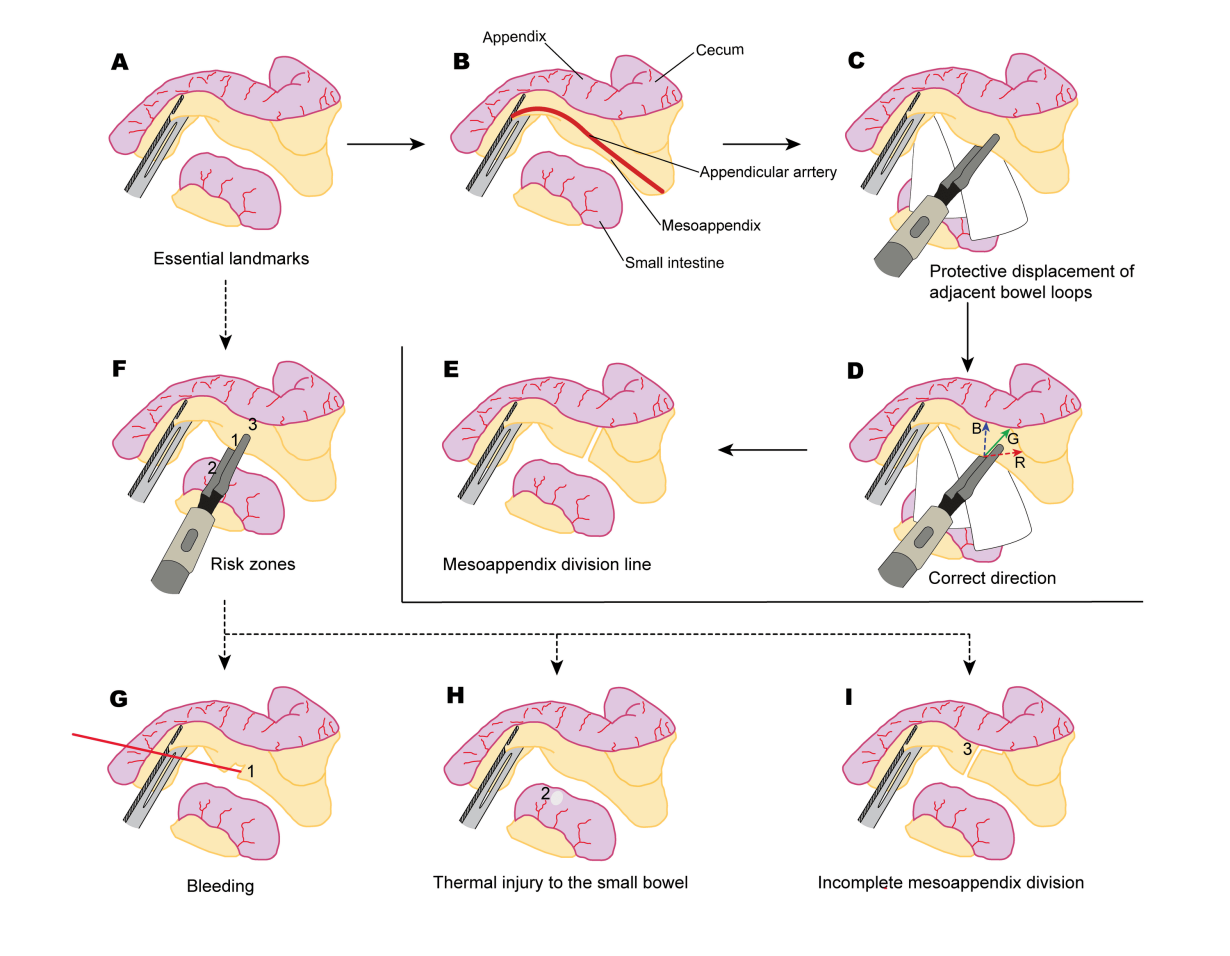
Stepwise illustrated surgical reflection framework demonstrating mesoappendix division and appendiceal artery control during laparoscopic appendectomy. Image credit: Loc H. Tran. Schematic illustration created by the author using Adobe Illustrator; not previously published.

The illustration process consisted of four structured steps (Figure 1), progressing deliberately from anatomical orientation to risk-based evaluation. To begin with, in step 1 (Figure 1A), simplified anatomical mapping was performed to establish spatial orientation using only essential landmarks, thereby reducing visual noise while clearly defining the appendix, mesoappendix, adjacent small bowel, and key vascular structures. Based on this anatomical foundation, step 2 (Figure 1B) highlighted critical structures and potential danger zones so that operative risks could be recognized before technical execution was introduced. Building upon this clarified spatial context, step 3 (Figures 1C-1E) demonstrated the appropriate operative strategy: protective displacement of adjacent bowel loops to maintain a safe working space (Figure 1C), identification of the correct mesoappendix division direction indicated by a solid green arrow, while dashed arrows illustrate unsafe trajectories; a blue dashed arrow (B) indicating an incorrect trajectory toward the appendiceal body and a red dashed arrow (R) indicating a hazardous direction toward the ileal mesentery (Figure 1D). These color-coded labels (B for blue, R for red) were added to ensure interpretability even in grayscale reproduction. This is followed by controlled energy application along the intended mesoappendix division line with secure appendiceal artery control (Figure 1E).

In contrast, step 4 illustrated improper technique and its consequences through three representative error patterns. Figure 1H demonstrates bleeding resulting from premature division of the mesoappendix without adequate coagulation or vessel control. Figure 1G illustrates unintended thermal injury to the adjacent small bowel when protective displacement is not maintained. Figure 1I shows an incomplete or excessively extended mesoappendix division caused by poor planning of the operative line. Through this structured progression and contrast between correct and incorrect techniques, the sequence integrates anatomical understanding, operative direction, bowel protection, and risk awareness into a coherent and reproducible reflective workflow.

Stepwise illustration of a simple anatomical structure

Illustrations can be generated using vector or bitmap graphics. The figures in this report were created using Adobe Illustrator (Adobe Inc., San Jose, CA, USA).

**Figure 2:**
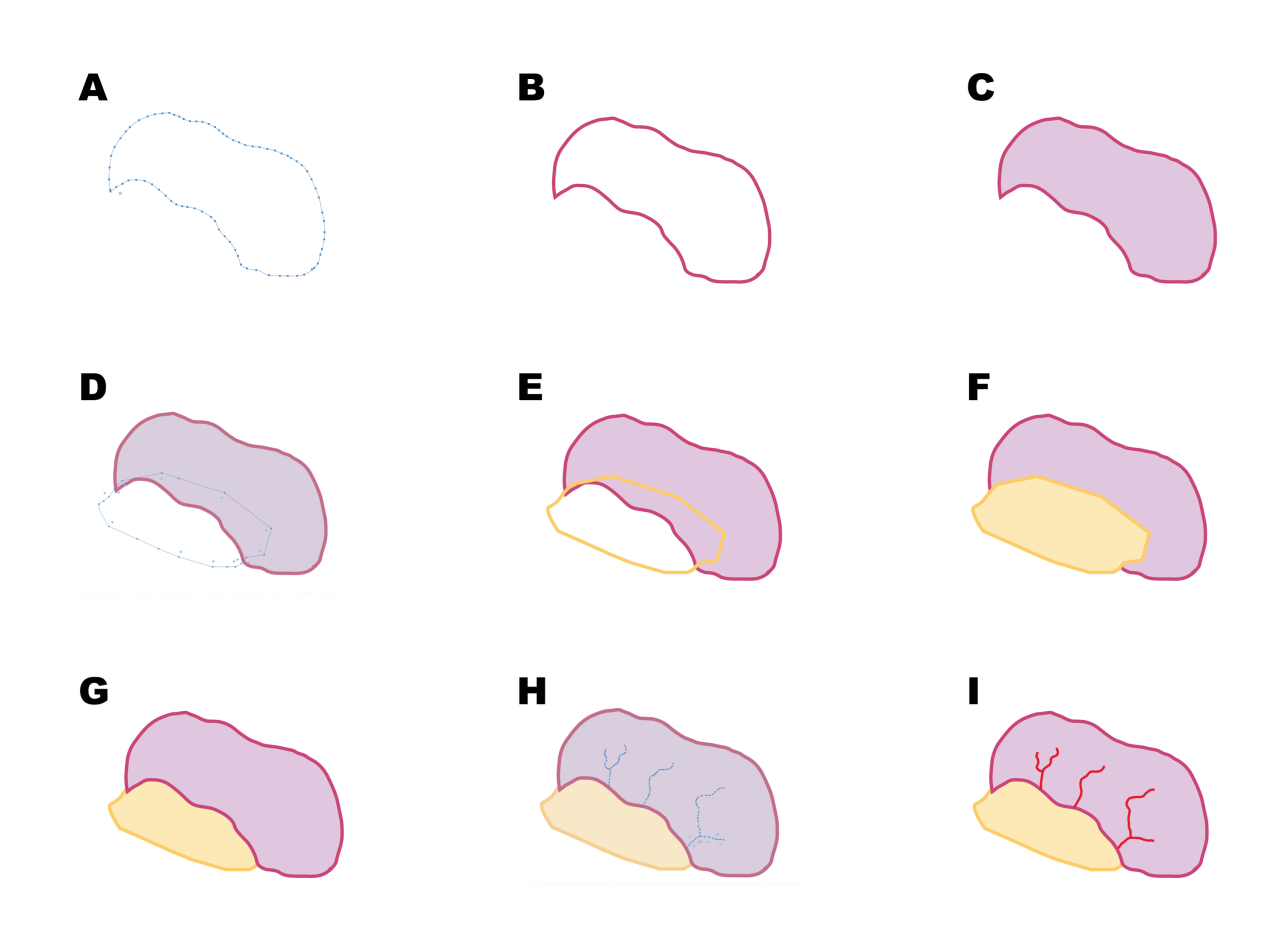
Stepwise illustration of a small bowel loop. Image credit: Loc H. Tran. Schematic illustration created by the author using Adobe Illustrator; not previously published.

Figure 2 illustrates the stepwise vector-based construction of a small bowel loop. In Figure 2A, anchor point tools are used to create the outline of the bowel loop. With the contour established, Figure 2B defines the boundary using stroke color, and Figure 2C adds fill color to establish the base form. The construction then proceeds in Figure 2D, where anchor points are used to create the mesentery as a separate object. In Figures 2E, 2F, stroke and fill are applied sequentially to complete the mesenteric structure. Figure 2G adjusts object arrangement to position the mesentery beneath the bowel loop, allowing controlled overlap and creating anatomical continuity using a simplified method suitable for beginners working with drawing tools. The process continues in Figure 2H with the construction of the superficial vascular elements using anchor points and concludes in Figure 2I with the color application to these vascular structures. The sequence reflects a structured workflow based on progressive object construction, contour definition, color application, and object organization to produce a clear anatomical illustration.

## Discussion

Technical imitation alone does not guarantee safe surgical practice [1]. In the early phase of training, trainees understandably focus on reproducing the hand movements demonstrated by their supervisors. Yet operative safety depends as much on judgment as on dexterity. Anticipating risk, recognizing vulnerable anatomy, and deciding when to proceed or pause are cognitive skills that are less visible than technical maneuvers. If these elements are not made explicit, trainees may reproduce form without fully understanding intent.

The difference between trainees and experienced surgeons, therefore, extends beyond manual skill [1]. Experienced surgeons constantly interpret subtle cues such as tissue resistance, vascular proximity, and spatial orientation while adjusting traction and energy use in real time. Much of this reasoning remains internal and is not always verbalized during routine teaching. A structured reflective method may help bring this reasoning into focus and provide trainees with a framework for analyzing what they are doing and why.

The PRAI model was designed as a practical scaffold for this purpose. By asking trainees to clarify the purpose of each step, identify potential risks, describe the chosen approach, and document the step visually, the framework encourages a shift from descriptive recall toward analytical understanding. Drawing, even in simplified form, requires deliberate selection of essential structures and conscious recognition of areas at risk [3]. Over time, this process may support clearer mental models and more consistent awareness of operative hazards.

This method may be particularly helpful in minimally invasive procedures. In operative settings, the surgical field often contains blood and surrounding tissues that obscure visualization [2]. In laparoscopy, some structures may also lie partially outside the camera’s field of view, further limiting spatial orientation and overall awareness. Simplified illustrations can help clarify essential reference points and spatial relationships that may be less apparent during the live procedure. In this context, illustration serves as a practical tool to support structured thinking.

This report illustrates the model through a single procedural example. Its impact on learning outcomes has not yet been evaluated and requires formal study. Future research should examine measurable educational outcomes and consider adaptation across different training environments.

Future studies may assess the educational impact of the PRAI framework using validated evaluation frameworks, such as the Objective Structured Assessment of Technical Skills (OSATS) domains [6], including tissue respect, instrument handling, and procedural flow, along with structured surgical error taxonomies (for example, wrong dissection plane or improper energy use). Additional measures could involve documenting near-miss events, such as bleeding due to premature vessel division or possible thermal injury to adjacent bowel; measuring the time required to identify key anatomical landmarks (e.g., the appendiceal artery); and applying cognitive workload instruments like the National Aeronautics and Space Administration Task Load Index (NASA-TLX) [7].

## Conclusions

Operative safety requires more than technical execution; it depends on the ability to recognize risks that may not be immediately visible. The PRAI-based illustrated reflection provides a simple framework to connect purpose, risk, and operative strategy. By reconstructing surgical steps visually, trainees may strengthen anatomical orientation and clarify operative reasoning. Illustrated reflection does not replace formal training, but may enhance the integration of experience and understanding. In this way, seeing the unseen becomes a trainable aspect of surgical thinking.
